# Clinical efficacy of nucleoplasty for uncontained lumbar disc herniation: a retrospective study

**DOI:** 10.1186/s12891-023-07120-3

**Published:** 2024-01-02

**Authors:** Yong Cheol Choi, Jong Hun Seo, Pius Kim

**Affiliations:** Department of Neurosurgery, College of Medicine, Chosun University, Gwangju, Korea

**Keywords:** Lumbar disc herniation, Uncontained disc herniation, Lumbar radiculopathy, Ruptured disc, Percutaneous lumbar nucleoplasty, Radiofrequency

## Abstract

**Background:**

There are insufficient in-depth studies on whether percutaneous lumbar nucleoplasty (PLN) is effective and safe for the treatment of uncontained lumbar disc herniation (ULDH). This study aimed to investigate the clinical efficacy of PLN on radiating leg pain caused by ULDH.

**Methods:**

Patients who underwent PLN for ULDH and met the inclusion criteria between June 2018 and July 2022 were included. Clinical outcomes were evaluated using the numeric rating scale (NRS) for radiating pain preoperatively; at 1 day, 1 week, and 1 month postoperatively; and at the last follow-up. Patient satisfaction was assessed using MacNab criteria.

**Results:**

Forty-one patients were enrolled. The mean age was 50.2 years (range 24–73 years). The mean and standard deviation of the preoperative NRS in 39 patients with radiating pain was 9.0 ± 1.2. The NRS scores at 1 day, 1 week, and 1 month postoperatively and at the last follow-up were 4.6 ± 3.2, 3.6 ± 3.3, 2.9 ± 3.2, and 1.4 ± 2.0, respectively, showing significant improvement (all, *p* < 0.001). The number of patients (percentage) with excellent or good satisfaction according to the MacNab criteria was 29 (70.7%). Major complications were not observed. Three patients underwent additional surgery after PLN because of persistent radiating pain.

**Conclusions:**

PLN is a safe and feasible treatment option for ULDH. Treatment outcomes were favorable on average; however, the lack of consistency was a drawback.

## Background

Lumbar disc herniation (LDH) is one of the most common spinal diseases in clinical practice. LDH levels vary among pathological subtypes. The classification system differs slightly among authors, and a definitive system has not yet been established [1–3]. Depending on the pathological type, symptoms, such as lower back pain, referred pain, or radiating pain in the lower extremities, may manifest differently. These symptoms are the most important factors in determining the treatment strategy for spine therapists. LDH can be pathological or undetectable. Contained lumbar disc herniation (CLDH) indicates that the displaced nucleus pulposus is covered with annulus fibers or the posterior longitudinal ligament, whereas uncontained lumbar disc herniation (ULDH) represents the absence of such a covering [4, 5]. Because ULDH is often accompanied by abrupt and severe lower-extremity pain or neurological deficits, it is generally not expected to improve with conservative treatment alone. Thus, it is recommended that the nucleus pulposus be removed and that direct neural decompression be achieved through surgery [6].

Percutaneous lumbar nucleoplasty (PLN) is a minimally invasive procedure that uses radiofrequency (RF) energy to remove nuclear material, reduce pressure in the disc space or herniated disc, and alleviate inflammatory mediators to improve LDH symptoms [7–11]. This technique has the significant advantage of being noninvasive, and several authors have shown that, if an appropriate indication is selected, both good clinical outcomes and rapid recovery can be achieved simultaneously [12, 13]. As mentioned in a review, CLDH is thought to be an appropriate indication for PLN, whereas ULDH generally is not [14]. However, there are insufficient in-depth studies on whether PLN is effective and safe for the treatment of ULDH. To answer this question, we collected and analyzed the clinical results of patients treated with PLN for ULDH and investigated its applicability.

## Methods

### Ethics statements

Before initiating the study, which aimed to investigate the clinical efficacy of PLN on radiating leg pain caused by ULDH, the Institutional Review Board approved the study protocol.

### Study design and patients

Between June 2018 and July 2022, 49 patients with ULDH underwent PLN performed by a single neurosurgeon, who was the corresponding author of this study. A comprehensive review of the patients’ medical records and radiologic images was conducted to identify patients who met the following inclusion criteria: (1) ULDH at a single level; (2) the presence of radiculopathy including radiating leg pain or motor weakness with corresponding lumbar disc herniation exhibiting root compression; (3) absence of other disc levels potentially causing radiculopathy in the lumbar spine; (4) absence of other lesions potentially causing pain or motor weakness on the lower extremities outside the lumbar spine; and (5) a minimum follow-up period of 3 months after the procedure. The exclusion criteria were (1) broad-based bulging disc herniation lacking apparent root compression; (2) contained lumbar disc herniation with the nucleus pulposus covered by the annulus fibrosus or posterior longitudinal ligament; (3) inadequate information within the medical records for analysis; (4) discogenic axial pain without evidence of lumbar radiculopathy in the medical records; and (5) indeterminate correlation between leg pain and radiologic findings.

### Data collection

Clinical outcomes were evaluated based on preoperative and postoperative pain improvement using a numerical rating scale (NRS) score and patient satisfaction, as determined by the MacNab criteria. NRS scores were collected preoperatively; at 1 day, 1 week, and 1 month postoperative; during the final follow-up. The MacNab criteria appraise patient satisfaction after treatment with ratings of excellent, good, fair, or poor. The time required for the NRS score to decrease to 2 and the patients’ return to work were also investigated. The presence of preoperative lower limb muscle weakness and extent of postoperative improvement were also assessed. Improvement was categorized as significant, slight, or no. Furthermore, postoperative complications and subsequent surgeries for persistent pain were examined.

### Surgical method for PLN

The patients were placed on the operating table in the prone position after preoperative antibiotic administration. A pillow was placed beneath the abdomen to facilitate flexion of the lumbar spine. Upon sterilization of the skin at the surgical site, we determined the trajectory and skin entry point 10–12 cm from the midline using fluoroscopic guidance. Local anesthesia was administered via a lidocaine injection along the approach path, extending from the skin to the deep tissue layers. An introducer needle, 1.8 mm in diameter and 19 cm in length, was advanced into the disc space through the Kambin triangle, an area delineated by the medial border of the nerve root, the lateral border of the superior articular process of the lower vertebra, and the lower endplate of the disc. Once the needle tip reached the nucleus pulposus, we inserted the device into the disc space via the introducer needle, and the radiofrequency (RF) probe was navigated toward the herniated disc segment under fluoroscopic visualization. Plasma energy was delivered around the probe to the target and surrounding region. Furthermore, the RF probe traced the migrated disc material (either upward or downward) that had entered the spinal canal, and we applied plasma energy while cautiously avoiding damage to the neural structure. The plasma produced by the RF probe situated within the nucleus pulposus inside the spinal canal elevates the temperature of the adjacent tissues encompassing the nerve roots, resulting in the patient experiencing a sensation of heat in the affected lower extremities. Plasma generation was briefly halted for a few seconds, followed by slight retraction of the RF probe and subsequent reapplication. In cases where the patient reported a recurrent sensation of heat in the lower extremities, the procedure was repeated. This method was previously presented in another publication by the same author as the “stepping back technique” and has been implemented in the lumbar spine [15]. Upon completion of all steps, the devices were withdrawn from the patient and the procedure was terminated. Patients were discharged within 1 week, with instructions to refrain from excessive activity for a minimum of 2 weeks.

### Statistical analysis

Discrepancies in NRS scores between the preoperative measurements and each postoperative assessment were analyzed using the paired t-test. Statistical analyses were conducted using R Statistical Software (version 4.2.2; R Foundation for Statistical Computing, Vienna, Austria). Statistical significance was set at p < 0.05 in all evaluations.

## Results

### Patient characteristics

A total of 41 patients (24 men and 17 women) met the inclusion criteria and were included in the analysis. The average age of the participants was 50.2 years, with an age range of 24–73 years. The average follow-up duration after the procedure was 29.4 months (range, 3–53 months). Regarding the frequency distribution by level, L4–5 was the most common, with 23 cases, followed by L3–4 in 9 cases, L5–S1 in 6 cases, L2–3 in 2 cases, and L1–2 in 1 case. Prolapsed nuclear pulposus migration occurred in an upward direction in 9 cases, downward in 24 cases, and not at all in 8 cases. Of the 41 patients, 14 presented with lower extremity motor weakness related to the affected nerve root preoperatively, and 2 of these patients did not experience radiating pain. This information is summarized in Table 1.

**Table 1:** Patient characteristics

Characteristics		Value
Age (years)		50.2 (24–73)
**Sex**		
	Male	24
	Female	17
**Disc level**		
	L1-2	1
	L2-3	2
	L3-4	9
	L4-5	23
	L5-S1	6
**Migration of disc fragment**		
	Upward	9
	Downward	24
	No migration	8
**Duration of symptom**		
	≤ 1 week	15
	> 1 week to ≤ 1 month	19
	> 1 month	7
**Onset pattern of symptom**		
	Sudden	31
	Gradual	10
**Grade of muscle strength**		
	V	27
	IV	9
	III	5
	II or below	0
Follow-up period (months)		29.4 (3–53)

Categorical values are presented as number, and numeric values are presented as average and range

### Clinical outcomes

**Fig. 1:**
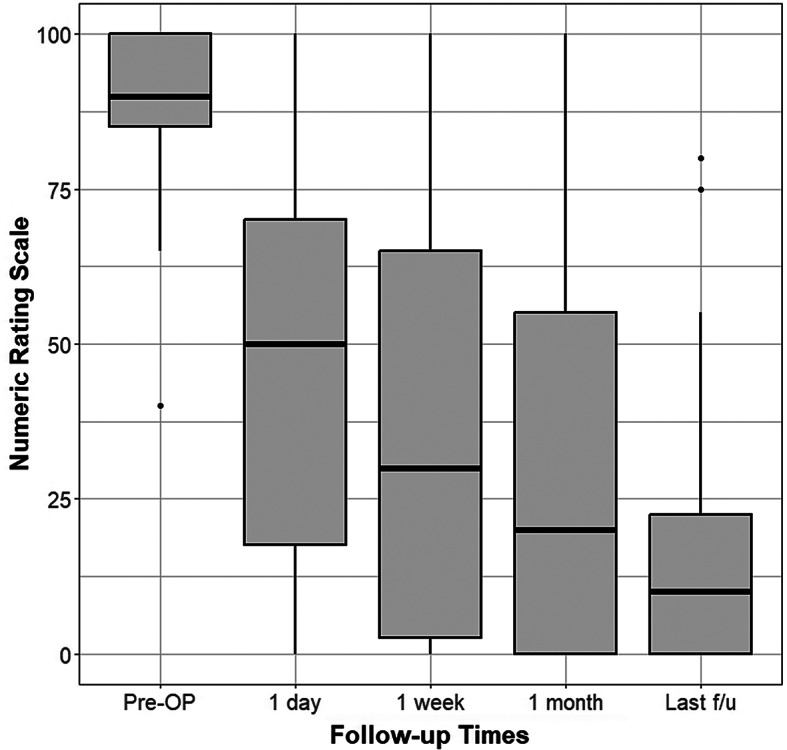
Boxplot illustrating the distribution of lower extremity radicular pain scores. The scores were measured using the numeric rating scale and are shown across the preoperative and postoperative follow-up periods. *Pre-OP* preoperative, *f/u* follow-up

**Fig. 2:**
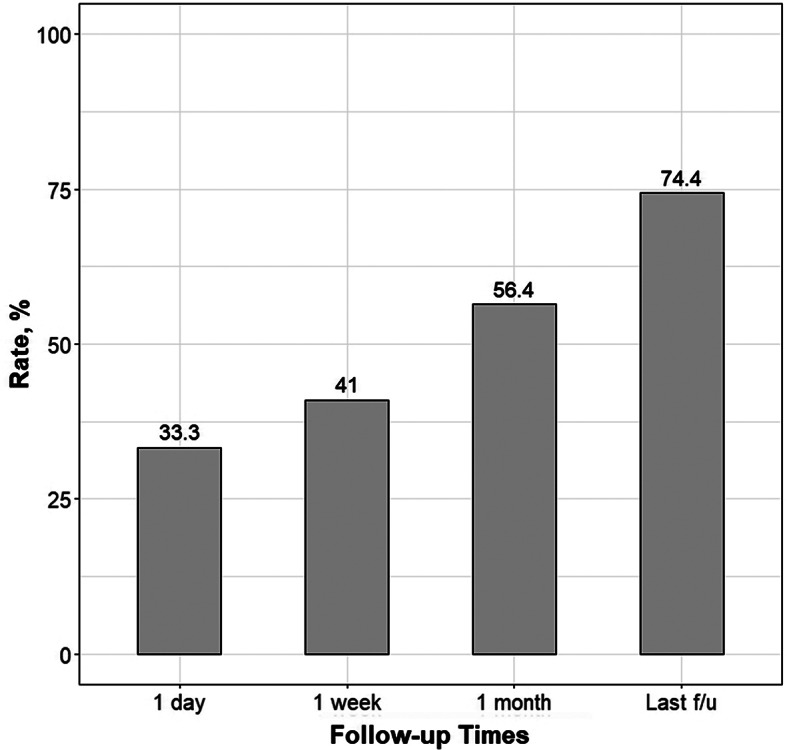
Barplot depicting the proportion of lower extremity radicular pain scores that decreased to ≤2. The scores were measured using the numeric rating scale throughout the postoperative follow-up periods. *f/u* follow-up

For the 39 patients experiencing radiating pain, the mean ± standard deviation preoperative NRS score was 9.0 ± 1.2. Postoperative NRS scores at 1 day, 1 week, and 1 month postoperatively and at the final follow-up were 4.6 ± 3.2, 3.6 ± 3.3, 2.9 ± 3.2, and 1.4 ± 2.0, respectively, demonstrating a statistically significant reduction in pain compared to preoperative values (all, *p* < 0.001; paired t-test). The results are presented in Table 2 and Fig. 1. At 1-month postoperatively, 22 of the 39 patients (56.4%) had NRS scores of ≤2 (Fig. 2). Of the 14 patients presenting with motor weakness, 11 reported considerable improvement at the final follow-up, whereas the remaining patients noted slight improvement. Within a month after the operation, 29 of the 41 patients (70.7%) resumed work. Based on the MacNab criteria, 29 of the 41 patients (70.7%) reported excellent or good satisfaction, whereas 12 patients experienced fair or poor satisfaction. No major postoperative complications were observed. Three patients required subsequent surgery after PLN because of persistent radiating pain.

**Table 2:** Decrease in radiating pain (numeric rating scale score) at the serial follow-up time points

	Mean ± SD	p-value	No. of NRS score of ≤ 2 (rate, %)
Pre-OP	9.0 ± 1.2	.	
1 day	4.6 ± 3.2	< 0.001	13 (33.3)
1 week	3.6 ± 3.3	< 0.001	16 (41.0)
1 month	2.9 ± 3.2	< 0.001	22 (56.4)
Last f/u	1.4 ± 2.0	< 0.001	29 (74.4)

*Pre-OP* preoperatively, *SD* standard deviation, *f/u* follow-up

### Illustrative case

**Fig. 3:**
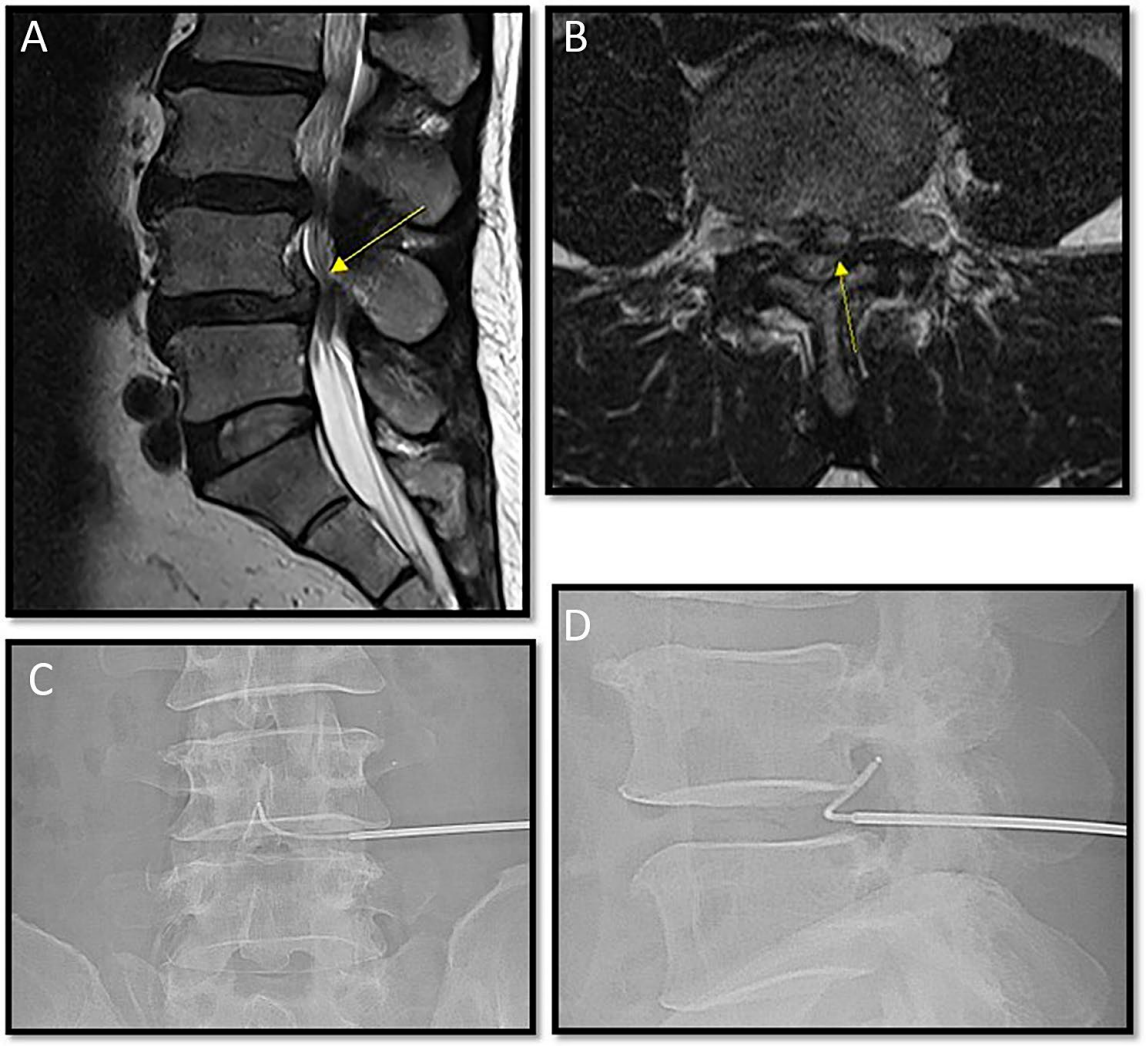
Magnetic resonance imaging (MRI) and intra-procedural fluoroscopy images of a 65-year-old man (case 1). (**a**, **b**) Sagittal and axial lumbar MRI T2-weighted images reveal spondylolisthesis of the fourth lumbar spine and uncontained lumbar disc herniation characterized by upward migration of the nucleus pulposus due to fibrous annulus rupture at the fourth to fifth lumbar intervertebral discs. The displaced nucleus pulposus is situated in the left paracentral direction, causing compression of the dural sac and the left fourth lumbar nerve root. (**c**, **d**) During percutaneous lumbar nucleoplasty performed with the patient under local anesthesia, fluoroscopy demonstrates successful access as the radiofrequency (RF) probe navigates through the torn opening of the fibrous annulus and follows the upwardly displaced disc fragment deep within the spinal canal. RF plasma was administered from this location to the interior of the disc for approximately 10 min

A 65-year-old man visited our hospital after experiencing sudden onset of lower back pain and continuous left lower extremity radicular pain radiating to the toes for 3 days prior. Prior to presenting at our hospital, the patient underwent pharmacological management and nerve block therapy at a local medical facility; however, these interventions failed to provide sufficient pain relief. The intensity of the lower extremity radicular pain was rated at 7 on the NRS, and a positive straight leg raise test result was observed on the left side. The patient’s lower-extremity strength and sensory function were normal. Lumbar magnetic resonance imaging performed at admission showed mild spondylolisthesis of L4 ULDH and upward migration at the L4–5 intervertebral discs, with left fourth root compression due to the disc fragment (Fig. 3a, b). The patient underwent PLN under local anesthesia on the following day. The RF probe was positioned well on the upwardly migrating disc fragment, and RF plasma was applied for approximately 10 min from this location to the disc interior (Fig. 3c, d). The stepping-back technique was used to prevent thermal injury to the neural structures. The procedure was successfully completed without any adverse events. The patient was transferred to the ward and, after a day of bed rest, was discharged the day after the procedure. His pain decreased to an NRS score of 1 at 1 day after the procedure, and it further reduced to an NRS score of 0 at 1 week later. The patient reported returning to normal work duties within a week after the procedure. At the last follow-up 25 months after the procedure, the patient reported mild discomfort with an NRS score of 1.

## Discussion

ULDH, also known as ruptured disc herniation, occurs when the annulus of the disc and the posterior longitudinal ligament tear, leading to leakage of the nucleus pulposus. Leakage compresses the nerve roots, resulting in acute pain in the lower extremities, hindered mobility, and diminished muscle strength. In some cases, the nucleus pulposus migrates within the spinal canal, making surgical resection challenging depending on the degree of migration and quantity of the nucleus pulposus. Over time, spanning a few weeks or months, the nucleus pulposus may be naturally absorbed, leading to symptom improvement. However, pain and neurological deficits often persist and necessitate surgical intervention. Previously, microscopic discectomy was the predominant surgical approach. However, contemporary spine surgeons are increasingly focusing on endoscopic spine surgery (ESS). Ongoing advancements in minimally invasive surgical methodologies have broadened the applicability of endoscopic techniques irrespective of the direction and extent of ULDH migration. As technology progresses and complication management is enhanced, ESS yields remarkable clinical outcomes, superseding conventional techniques and exhibiting immense potential for future growth [16–19].

Although ESS has been making great strides, PLN did not become a major focus of spinal surgeons and was only recognized as a treatment for discogenic pain or CLDH. In fact, very few patients with ULDH have been treated with PLN; therefore, there is a lack of concrete research data. It is generally thought that it is impossible to treat ULDH with PLN, and the use of RF plasma in the spinal canal is considered risky because it can cause thermal damage to the neural structures. Some authors have argued that ULDH is not an indication for PLN [12, 20].

However, in this study, the use of an RF probe to access a migrated disc fragment within the spinal canal was not extremely dangerous. Using the stepping back technique, we did not encounter any cases of thermal damage to the neural structures due to PLN, confirming that PLN for ULDH is neither dangerous nor impossible. This study’s results showed that the treatment effect was positive, as evidenced by the rate of improvement in lower extremity pain and muscle weakness and the rate of return to work in all patients. These results were achieved by applying RF plasma percutaneously with the patients under local anesthesia for 10 min without surgery, which translates into a more efficient treatment than surgical treatment when considering the length of hospitalization, pain at the treatment site, recovery time, and incidental costs.

When performing PLN, we aimed to achieve as complete access to the RF probe as possible, even for disc fragments that had migrated upward or downward, because we believe that RF plasma should be applied to all disc fragments within the spinal canal to achieve maximum nerve compression relief with PLN. Therefore, the case described herein is an ideal one in which the treatment goal was to achieve a good therapeutic effect quickly and without complications through complete access. However, there were also cases in which complete access to the RF probe was difficult owing to anatomical circumstances, such as reduced disc height, but there were still some therapeutic effects. Thus, we pondered the role of generating RF plasma from the nucleus pulposus inside the disc, reaching the opening of the fibrous annulus or within the spinal canal around it.

The pain-inducing role of inflammatory mediators has been suggested by many authors as a possible mechanism of pain in the disc [21–24]. Neovascularization and nociceptive nerve endings at the foci in the annulus fibrosus and endplates are also major causes of disc pain [25, 26]. The resulting pain can manifest as pain in the lower extremities [27, 28]. ULDH develops after years of chronic damage to the annulus fibrosus due to degenerative changes, when pressure from within the disc causes the nucleus pulposus to break through the last boundary of the annulus fibrosus and burst into the spinal canal. The fibrous annulus tears rapidly, triggering an acute inflammatory response, and nociceptive nerve endings are severely irritated by the inflammatory response and pressure, which can cause acute low back and referred pain in the lower extremities. This is another pathological mechanism that causes pain in the lower extremities, in addition to radiating pain from nerve root compression caused by the ruptured nucleus pulposus, which simultaneously causes lower extremity pain. RF plasma removes inflammatory mediators and cauterize nociceptive nerve endings as a therapeutic mechanism, and it appears to play a role in treating the aforementioned pathological conditions through the same mechanism [8–11]. It is also thought that by lowering the pressure within the disc, the pressure transmitted through the annulus fibrosus opening into the spinal canal can be somewhat reduced, and by removing the disc fragments adjacent to the opening, the nerve root pressure can be reduced to some extent. One important advantage is that by removing some of the nucleus pulposus inside the disc, we may reduce the likelihood of recurrence in the future, where additional nucleus pulposus may ooze out. Additionally, this study found that disc fragments that traveled further through PLN could be tracked and cauterized.

However, when we closely examined the study’s clinical results, we found a critical flaw in PLN. Although the mean preoperative and postoperative pain scores were significantly reduced, the distribution of the scores was widely skewed around the mean with a large standard deviation, as shown in Fig. 2. Furthermore, while some patients experienced most of their pain reduction immediately after the procedure, there were extreme values, with some patients showing no or only a very slight reduction in pain scores from preoperatively to postoperatively. Three patients were dissatisfied with their symptomatic improvement and ultimately underwent surgical intervention, resulting in increased pain duration, healthcare costs, and treatment duration. This finding suggests that PLN for ULDH may be effective on average but that it has significant limitations in terms of consistency and reliability. Can we confidently recommend PLN to patients presenting to the emergency department with severe lower extremity radiation pain due to ULDH? Most spine surgeons know from clinical experience that surgical treatment can produce immediate pain relief in most cases and can be expected to be consistent; this is well documented in the literature [29–31].

The strength of this evidence is limited by the fact that this study was retrospective and included only a small number of patients at a single institution. In the future, large-scale studies with a design that can provide stronger evidence are needed.

## Conclusions

PLN is a safe and feasible treatment option for ULDH. Treatment outcomes were favorable on average; however, the lack of consistency was a drawback. Future developments in technology and equipment will need to address these shortcomings if the treatment is to advance. Further research is needed on transdiscal approach techniques, such as PLN for ULDH.

## Data Availability

The data is available upon request, in consultation with the corresponding author (PK).

## Abbreviations

LDH: Lumbar disc herniation
CLDH: Contained lumbar disc herniation
ULDH: Uncontained lumbar disc herniation
PLN: Percutaneous lumbar nucleoplasty
RF: Radiofrequency
NRS: Numerical rating scale
ESS: Endoscopic spine surgery
